# Language attitudes of Generation Z in responding to teacher questions in learning interactions

**DOI:** 10.1590/2317-1782/e20250098en

**Published:** 2025-09-04

**Authors:** Imam Suyitno, Ary Fawzi, Heni Dwi Arista

**Affiliations:** 1 Indonesian Letters Department, Universitas Negeri Malang - Malang, Indonesia.; 2 Indonesian Department, Universitas Brawijaya - Malang, Indonesia.

**Keywords:** Language Attitude, Generation Z, Teacher Questions, Student’s Responses, Learning Interaction

## Abstract

**Purpose:**

This study aimed to examine the language attitudes of Generation Z in responding to teacher questions in the classroom interaction. The focus of the study was the problems of the diversity of language forms and strategies, the influencing factors, and their implications for learning dynamics.

**Methods:**

This study used a qualitative approach, and the data collection was conducted through observation and semi-structured interviews. Researchers observe and record students’ speech and its contexts related to their responses to teacher questions during Indonesian language, arts and culture, and mathematics lessons. After class activities, the researcher conducted semi-structured interviews with students to explore causal factors related to language habits and the use of digital social media. The researchers also interviewed teachers to explore the impact of these speeches use in learning interaction.

**Results:**

The study reveals that Generation Z students respond to teachers' questions frequently using informal Indonesian, slang, regional languages, and mixed language styles, reflecting their preference for familiarity and self-expression over strict formality in classroom interactions. Students employ diverse language strategies to respond to teachers’ questions, such as short answers, humor, metaphoric appeals, and challenges. The study found that Generation Z's speech in responding to teacher questions is influenced by social media exposure, informal teacher-student relationships, and socio-cultural backgrounds, leading to a preference for informal and digital-influenced language in the classroom and social interactions.

**Conclusion:**

This study concludes that Generation Z's language attitudes in the classroom are different from previous generations in responding to teacher questions. Generation Z's language attitudes are influenced by language in digital social media and local culture, fostering egalitarian interactions while posing challenges in maintaining academic formality.

## INTRODUCTION

Language attitudes in learning interactions can influence and create a conducive and effective learning atmosphere. Students' language attitudes need to be developed through natural language training to achieve the goals of language teaching, namely the ability to use language in various interaction contexts^(1)^. In education, using polite language by teachers and students can prevent conflict and increase respect for each other. The application of language attitude in interactions between teachers and students functions to express information, ask for permission, give orders politely, and convey criticism constructively^(2)^. This shows that language attitudes play an important role in maintaining harmony and the effectiveness of the learning process.

Classroom interaction is a learning activity that reflects the relationship between teachers and students. It affects students' success in achieving learning experiences. In learning, teachers often build effective interaction through questions and answers. Teacher questions function as a vehicle to measure student understanding and encourage students to think critically, argue, and actively participate in class activities^(3)^. Applicable and analytical teachers’ questions encourage students to be involved in class discussions^(4)^. The questions can be a bridge to encourage students to think critically and provide responses. In the learning context, teachers should be sensitive to students' language attitudes to anticipate the constraints of learning activities. Students' language style, which is open and informal responses to the teacher's questions, can be language teaching materials to direct students' conversations into a more structured academic domain.

Generation Z students grew up in an environment filled with social media and fast-paced communication technology, shaping their interaction outside formal contexts. The use of informal language, slang, and digital elements such as emojis, acronyms, and abbreviations has become part of their daily lives^(5)^. Language attitudes that do not conform to formal classroom communication can potentially cause miscommunication or affect teachers' perceptions of students^(6)^. This kind of language attitude can reduce the quality and depth of their understanding in class discussions, especially when faced with questions that require critical thinking or more complex arguments^(7)^.

In the current learning context, teacher questions often get unexpected language responses from students due to digital technology influences. The presence of digital technology in everyday life has formed ways that are shorter and more straightforward communication, and sometimes even uses language elements that are unusual in formal contexts^(8)^. The popular language and expressions used on social media influence students' language attitudes in responding to teacher questions^(9)^. This phenomenon can affect learning activities concerning teachers' perceptions of students, as well as students' perceptions of the authority and role of teachers. Therefore, teachers must understand students' speech styles in learning interactions to minimize the risk of miscommunication that can hinder the learning process^(10)^. Teachers’ understanding of students' language attitudes can help teachers determine adaptive teaching methods, create space for higher student engagement, and encourage more productive interactions in the classroom^(11)^.

Based on the description above, it can be summarized that language attitudes have an important role in creating effective learning interactions, especially in building polite, critical, and productive communication between teachers and students. In the context of generation Z, who are accustomed to informal digital communication, teachers need to understand students' speech styles and develop adaptive learning strategies to minimize miscommunication and optimize the achievement of learning objectives. Therefore, this study aims to examine the language attitudes of Generation Z in responding to teacher questions in the learning interaction, focusing on Generation Z's language forms, language strategies, the factors that influence the language attitudes of Generation Z. Furthermore, this study also explores how Generation Z's language attitudes in responding to teacher questions in learning interactions affect learning dynamics, including student engagement, understanding of the material, and communication barriers that may arise.

Previous research examining questioning strategies in classroom interactions indicates that teachers use rephrasing, simplifying, repeating, elaborating, and probing^(12)^. These strategies have various purposes based on the functions associated with each strategy. Students believe that the strategies improve their understanding of the target language material they are learning. In addition, communication in the classroom performance has a positive relationship with student learning outcomes^(13)^. Teachers use questions to encourage students to think critically. The difference between this study and the two previous studies is that this study is more in-depth in linking social, cultural, and technological factors that influence students' speaking attitudes and focuses more on the dynamics of interactions between students and teachers.

This research is significant for providing new insights into how Generation Z, who grew up with technological advancements, interacts in educational contexts. A deep understanding of their communication forms and strategies can help teachers adjust teaching methods to be more effective and relevant to students' communication. The findings of this study contribute to developing theories on communication in education and help reduce communication barriers between students and teachers in learning. Thus, this research is beneficial for developing pedagogy and improving students' learning experiences, especially when facing communication challenges related to changes in speech styles due to digital technology.

## METHODS

### Research design

This study used a qualitative approach to identify and analyze Generation Z’s language attitudes in responding to teacher questions in class. A qualitative approach provides space to explore phenomena holistically, understand context, and interpret data in a broader socio-cultural context^(14,15)^. This research has obtained approval from the ethics commission of Universitas Negeri Malang (UM), and a letter of approval No. Ref: 24.3.2/UN32.14/PB/2025, dated March 24, 2025. The data collection of this study was conducted during six meetings, with details: two meetings for observation and interviews for each class of Indonesian language, arts and culture, and mathematics. To ensure the adequacy of the data, the researcher extended the time to re-collect data through unstructured interviews with students and teachers.

### Research location and subjects

Researchers conducted the study at SMA at Malang, a private school whose students come from various areas with diverse social and cultural backgrounds. The subjects of this study were grade X and generation Z students, who have the same characteristics as students of the same age in other schools. This study also involved Indonesian language, arts and culture, and mathematics teachers who taught the subject to students as informants. The selection of these subjects is based on the reason that the three subjects have their strategic roles in developing students' critical, creative, and logical thinking skills. Indonesian language learning builds critical communication, arts and culture develop creative skills, and mathematics learning develops logical thinking skills.

### Data collection

The study data were verbal data about students' language in responding to teacher questions in classroom learning interactions. The data comes from learning interaction discourse collected through focused observation to record and note students' speech and the speech context that underlies it. The learning interactions realized in classroom conversations recorded in the audio and text transcripts are part of the research data source^(16)^. This study also involved three data source informants, namely Indonesian language, arts and culture, and mathematics teachers, who interacted with the students to understand their perspectives on the dynamics of the interactions.

Data collection was conducted through direct observation of classroom learning interactions, observing and recording how students responded to teacher questions, the variety of languages ​​used, and the teacher's reactions. The study following Spradley conducted observation naturally records student speech in the dynamics of classroom learning interactions^(17)^. In addition, researchers also conducted semi-structured interviews with students to explore causal factors related to language habits and the use of digital social media, and with teachers to explore their perceptions of students' language attitudes in responding to questions in class. This interview aimed to understand how students responded to teacher questions and the reasons behind their language choices^(18)^.

Researchers used observation sheets to record and document various language forms and strategies that emerged in interactions between students and teachers. Interview guidelines were compiled based on questions about language forms, language strategies, and influencing factors in classroom interactions. The interview guide facilitates researchers' interviews with students and teachers^(19)^.

### Data analysis

Data analysis was carried out inductively by following the following steps.

1. Data identification and classification: Data from interviews and observations were analyzed, identified, and classified using coding techniques. At this stage, this study codes parts of the text based on the focus of the research, such as language forms, language strategies, and factors that influence language attitudes^(19)^;
2. Data categorization: After coding, the study grouped data into emerging categories based on themes related to speech varieties, social media influences, and socio-cultural factors. This categorization allows researchers to identify patterns and relationships between existing factors^(20)^;
3. Thematic Analysis: Using thematic analysis, the researcher analyzed the main themes from the data, such as how students used informal language or slang and the factors that influenced their communication. This process helped identify common patterns in students' speaking attitudes and how these influenced the dynamics of classroom learning^(21)^;
4. Contextual Interpretation: After finding themes and communication patterns, researchers interpret the results in the socio-cultural context of the school environment and the community where the students live. At this stage, the researchers interpret influencing factors, such as social media, teachers-student relationships, and students' sociocultural backgrounds^(22)^.

### Validity of data

This study uses triangulation to ensure the validity of the data by comparing the results of interviews and observations. In addition, to ensure the validity of the findings, the researcher uses member checking, namely confirming the analysis results with the subject to ensure the interpretation is under their views^(23)^.

## RESULTS

This section presents research findings on Generation Z’s language attitudes in responding to teacher questions to unveil the dynamics of classroom learning interactions. The study highlighted students' language attitudes through language forms, language strategies, and influence factors of language attitudes. The findings are below.

### Generation Z language forms in responding to teacher questions in class

Observation notes on learning the Indonesian Language, Arts and Culture, and Mathematics in high school show that students respond to teacher questions using various forms of language. The examples below describe students’ language forms in responding to the teacher's question.

Example 1:

When the teacher explained the topic of writing anecdotes in the Indonesian language class, two students did not pay attention and joked with their friends. To reprimand them, the teacher gave a question to the naughtiest student, with the following question.

**Original Texts:**

Guru: AN, … coba jelaskan kepada temanmu, apa teks anekdot itu?

Siswa A: Kok aku se Bu yang ditunjuk suruh jawab? Apa ya Bu…?

Guru: Makanya, kalo dijelaskan itu jangan bergurau. Coba kamu, Bin?

Siswa B: Hah …., gak bisa Bu….

*English Translation:*

Teacher: AN, … please explain to your friend what an anecdote is?

Student A: Why am I to answer? What is it, Ma'am?

Teacher: That’s why, when explaining it, don’t joke. Try it, Bin?

Student B: Huh …., I can’t, Ma’am…

Example 2:

In art and culture learning in high school, the teacher explains the variety of arts in Indonesia and the regions of origin of the arts. The teacher uses the question-and-answer method to build active learning by involving students in class interactions. The teacher asks students about several topics.

**Original Texts:**

Guru: Coba sebutkan contoh kesenian khas daerah dan sebutkan dari daerah mana?

Siswa C: Gandrung dari Banyuwangi…. Bener kan Pak?

Siswa D: Reog dari Ponorogo.

Guru: Bagus…. Yang lainnya?

Siswa E: Tari Pendet, …. sik yo, dari mana ya? Kalo gak salah Bali ya Pak?

Siswa F: ada lagi Pak, tari Topeng…. dari Malang….

Siswa G: Ndik Madura onok lo tari Topeng….

*English Translation:*

Teacher: Try to mention an example of regional art and where the region is.

Student C: Gandrung from Banyuwangi…. Right, Sir?

Student D: Reog from Ponorogo.

Teacher: Good…. The others?

Student E: Pendet Dance, …. Sik yo, where is it from? If I’m not mistaken, Bali, right, Sir?

Student F: There’s another one, Sir, Mask Dance…. from Malang….

Student G: Ndik Madura onok lo Mask Dance….

Example 3:

The teacher was in class, waiting for her students to take the math test. When 10 minutes were left for work, a teacher asked the students the following questions.

**Original Texts:**

Guru: Waktu mengerjakan tinggal 10 menit. Sudah selesai apa belum? Apakah sulit soalnya?

Siswa H: dikit lagi, …. Sulit poll….

Siswa I: Waduh…. Jik akeh sing belum tak garap….

Siswa J: bantuin ta Pak …. biar cepet selesai.

Siswa K: Wis mumet aku, sak mariku ae

*English Translation:*

Teacher: There are only 10 minutes left to work on it. Have you finished it yet? Is the test difficult?

Student H: Just a little bit more, … It's so difficult….

Student I: Oh no…. If I haven’t done it yet, I won’t do it….

Student J: Help me, Sir! …. so it's finished quickly.

Student K: I’m so confused, let’s go

Regarding the language forms used by Generation Z, this study interviewed teachers about their responses to students' language attitudes. Interviews with teachers obtained the following information.

Quote 1: Indonesian Language Teacher's Response

I realized that the student’s language was not justified according to the rules of conversation in formal situations, to maintain a comfortable atmosphere and conducive learning motivation. However, I did not reprimand him. I understand the attitude of students who want familiarity with class communication. However, at the end of the lesson, I emphasized the importance of using the correct Indonesian for conversation in formal situations.

Quote 2: Arts and Culture Teacher's Response

In learning arts and culture, I emphasize understanding the content of the subject matter. I see students' speech in various languages to express comfort and create a more relaxed learning atmosphere. In class interactions, I emphasize polite speech and maintain ethics in communication.

Quote 3: Mathematics Teacher's Response

When giving math tests, I do not care about the language variety used in students' speech to respond to my questions. Several students use regional languages, while others use Indonesian in informal conversations. I consider these language varieties used to express their boredom because they face difficult questions.

### Generation Z language strategies in responding to teacher questions

Based on recordings of students' speech during Indonesian Language, Arts and Culture, and Mathematics learning, generation Z's language strategies can be found in responding to teacher questions in class. The results of the analysis are in the following examples.

Example 4:

In Indonesian language learning activities, the teacher presents teaching materials on writing anecdotal texts. The teacher begins his learning activities by using the lecture method to provide students with an understanding of anecdotal texts' meaning, characteristics, functions, and structure. After completing her explanation, the teacher wants to know the students' achievement by asking several questions.

**Original Texts:**

Guru: Coba siapa tahu, apa ciri-ciri teks anekdot itu?

Siswa A: Lucu Bu …

Siswa B: Menyindir

Guru: Apa cirinya yang lain, Ci?

Siswa C: Kalo pengertiannya, saya tahu, … Bu. Kisah yang lucu …

Siswa D: Tanyakan ke saya, fungsi teks anekdot Bu …

Guru: Iya, apa fungsinya Din?

Siswa E: Cerita yang membuat orang tertawa,… hahaha….

*English Translation:*

Teacher: Who knows? what are the characteristics of an anecdote text?

Student A: Funny, Ma'am.

Student B: Sarcastic

Teacher: What are the other characteristics, Ci?

Student C: The definition I know, Ma'am. It is a funny story.

Student D: Ask me the function of an anecdote text, Ma'am!

Teacher: Yes, what is its function, Din?

Student E: A story that makes people laugh. Hahaha

Example 5:

In the learning, the teacher of the Art and Culture subject, after explaining the material and asking and answering questions with students about the variety of traditional arts in Indonesia, gave students the task of writing down ten varieties of traditional arts in Indonesia and mentioning their regions of origin. After a few minutes had passed, the teacher greeted the students with the following questions.

**Original Texts:**

Guru: Sudah selesai apa belum mengerjakan tugasnya?

Siswa F: Sabar Pak, Belanda masih jauh….

Siswa G: Tugasnya kurang banyak Pak….

Guru: Menurut kalian, apa tugas itu sulit?

Siswa H: Tidak sulit, tapi buanyak….

Siswa I: Bukan core 2 duo …

*English Translation:*

Teacher: Have you finished your assignment or not?

Student F: Be patient, Sir! The Netherlands is still far away….

Student G: There aren't many tasks, sir...

Teacher: In your opinion, are the assignments difficult?

Student H: Not difficult, but there are too many….

Student I: Not core 2 duo …

During interviews with teachers, this study obtained information that many students in the class like humor and like to tease their friends. They use humor and sarcasm to avoid tension or divert attention from the topic's difficulty. Students use humor to disguise their ignorance of the material. They often give short and direct answers without too much explanation.

### Factors influencing generation Z speech in responding to teacher questions

Concerning data on the factors influencing Generation Z speech, this study interviewed three teachers teaching the subject class. The results of the interviews are in the following excerpts.

Quote 4: Information about student communication with teachers

We always create good relationships with students. When communicating in informal situations, we often use relaxed and informal language to build trust and personal closeness. A more intimate and egalitarian relationship with students allows students to feel more comfortable using informal language when communicating. However, this way of communicating is often brought by students in communication in the classroom learning.

Quote 5: Information about the student's background

Students who study at this school come from a community environment that is thick with the Javanese language. In everyday life, students generally use Javanese. Students communicate at home and in social environments using their regional language. They joke with friends in the school environment and the community using informal or regional language.

Quote 6: Information about social media

Our monitoring during recess can inform us that students prefer to play with their gadgets rather than play with their friends. Social media seems to play a significant role in shaping the communication style of the millennial generation. The use of social media has changed the way they communicate. Communication patterns on social media tend to be fast and use pop culture references, reflected in communication in and outside class.

## DISCUSSION

### Generation Z's language attitudes in responding to teacher questions

The research findings show that Generation Z’s language attitudes in responding to teacher questions in class show a variety of speech forms, ranging from formal to informal, slang, and mixed languages. Most students use informal language varieties, reflecting comfort in relaxed and familiar interactions, although less appropriate for the formal learning context. Teachers balance this familiarity with formal language use to increase students' awareness of formality in academic communication. These findings on the variety of language forms are categorized in Table 1. The table shows that only two students (D and F) use formal Indonesian. Meanwhile, nine students use informal Indonesian, slang, local regional language, and mixed language (informal Indonesian and local regional language).

**Table 1:** Categorization of Language Varieties from Student Speech

**Student Responses**	**Language Variety**	**Speech Data**
Student A	Mixed	*Kok aku se Bu yang ditunjuk suruh jawab? Apa ya Bu…?*
Student B	Informal language	*Hah …., gak bisa Bu….*
Student C	Slang	*Bener kan Pak?*
Student D	Formal language	*Reog dari Ponorogo*
Student E	Mixed	*sik yo, dari mana ya? Kalo gak salah Bali ya Pak?*
Student F	Formal language	*ada lagi Pak, tari Topeng…. dari Malang….*
Student G	Local language	*Ndik Madura onok lo tari Topeng….*
Student H	Informal language	*dikit lagi, …. Sulit poll….*
Student I	Mixed	*Waduh…. Jik akeh sing belum tak garap….*
Student J	Slang and local language	*bantuin ta Pak …. biar cepet selesai.*
Student K	Local regional language	*Wis mumet aku, sak mariku ae*

These findings show that the phenomenon reflects the desire of Generation Z to communicate familiarly and informally. They prioritize self-expression and familiarity, although sometimes they pay less attention to the context of formality. For this reason, teachers need to consider adapting communication methods that are more responsive to students' language styles, especially on digital platforms, without sacrificing learning objectives. In learning, teachers need to educate students about when to use formal language and when to use informal language so that they better understand the appropriate communication context.

The study findings correspond to the theory of intergenerational communication. The theory states that language use reflects an individual's social identity and comfort in a particular group^(24)^. Previous studies also indicate that students use informal language to express themselves in environments that they consider non-threatening^(25)^. This condition confirms that the informal language influenced by the dynamics of egalitarian relationships between teachers and students does not always correspond to the formal context of education.

The second finding, as in Table 2, shows that Generation Z used language strategies, including short speech, avoidance, challenges, humor, metaphorical allusions, direct speech, contradictory statements, and sarcasm. This language style is the habit of interacting on social media that demands fast, efficient, and often humorous responses. This phenomenon reflects a more egalitarian communication pattern between students and teachers, presenting challenges and opportunities for teachers to design teaching methods to respond to students' communication styles without sacrificing learning objectives.

**Table 2:** Categorization of Students' Language Strategies in Responding to Teacher Questions

**Students**	**Language Strategies**	**Indicators**
A	Short answer	Short answer *“lucu”*
B	Short answer	Short answer *“menyindir:*
C	Avoidance	Offering another question *“Kalo pengertiannya, saya tahu”*
D	Challenging	Challenge*“Tanyakan ke saya, fungsi teks anekdot Bu “*
E	Humorous	Answer and laugh*“hahaha….”*
F	Metaphoric appeal and satire	Begging and insinuating*“Sabar Pak, Belanda masih jauh”*
G	Direct statement	Direct sarcasm *“Tugasnya kurang banyak Pak”*
H	Contradictory statement	Using contradiction *“Tidak sulit, tapi buanyak”*
I	Metaphoric sarcastic	Insinuating using metaphors *“Bukan core 2 duo”*

The diversity of language strategies reflects the influence of communication habits on social media. Social media has changed the communication patterns of the younger generation to be faster, more direct, and often relaxed or humorous^(26)^. This phenomenon supports the previous study that explains that social media influences students' speaking styles and challenges teachers to balance familiarity and academic goals^(27)^. This condition challenges and demands that teachers develop more effective and adaptive communication methods in learning. Teachers need to be more sensitive in handling sarcastic and humorous responses and understand that these responses do not mean being impolite but reflect a communication style formed from everyday interactions in the digital world.

### The factors influencing generation Z's language attitudes on learning interactions

The factors that influence students’ language attitudes can be understood from the students' interview notes as in Table 3. The table shows that the factors influencing Generation Z's language attitudes include egalitarian teacher-student relationships, the habit of using regional languages ​​in everyday life, and the influence of interactions on social media. The influence of social media, relationships with teachers, and socio-cultural backgrounds significantly shape the language attitudes of Generation Z. These changes in communication patterns reflect students' adaptation to the ever-evolving digital and social environment. In such a context, teachers must understand these dynamics to create a more inclusive and adaptive learning environment while instilling the importance of communication ethics in various contexts.

**Table 3:** Students Interviews Note

Num	Questions	Answers
1	Do you often play around with gadgets?	Almost all students said “often”. Some even felt addicted
2	How long do you use gadgets in a day?	They found it difficult to determine the duration because they filled their time using gadgets
3	Do you use gadgets for studying or other purposes?	Most of them used gadgets for entertainment, but when they had an assignment, they used their gadgets to study.
4	What applications do you often use on your gadgets?	They most often used TikTok and YouTube. Several students used Instagram and WhatsApp and also read funny or silly news.
5	Do you like the language used in the applications you watch?	They liked the language because they felt cool, more fun, shorter, and easier to understand.
6	Do you often use the language of the applications you watch to communicate with friends?	They often imitated and used the language in social media to communicate with friends because they felt familiar and relaxed.
7	When communicating with teachers, do you also use social media language in the gadget applications you watch?	They answered that most of their teachers were fun and relaxed. They also didn't feel awkward speaking using informal language.
8	What language do you often use at home or in your neighborhood?	They most often used regional languages. Sometimes using slang when talking to their peers.

The relaxed social and cultural environment encourages students to use informal language even in formal learning situations. Factors such as egalitarian relationships between teachers and students, local language use, and interaction habits on social media are the main determinants of students' communication styles^(28)^. Furthermore, a social environment encourages relaxed communication by using informal language preferences^(29)^. This phenomenon influences language choices in formal learning situations, as students may carry these communication habits into academic settings, leading to a blend of informal and formal linguistic features^(30)^.

Students’ experiences of digital culture affect the dynamic of classroom learning. The experiences change the students’ way of learning and responding to the teaching materials. They tend to be critical and want an active role in the learning process, but they also often show a more relaxed and less formal attitude^(31)^. These conditions challenge teachers in managing the classroom and creating a conducive learning environment^(32)^. Teachers must understand how Generation Z characteristics adapt to conventional learning formats^(33)^. Therefore, teachers must develop adaptive teaching strategies according to students' learning styles to engage in learning interactions and motivation and achieve learning goals optimally^(34)^.

These findings emphasize the importance of understanding students' social and cultural contexts in designing inclusive learning strategies^(35)^. Teachers can use this understanding to integrate students' communication styles into learning to create a dynamic atmosphere while still paying attention to academic communication ethics^(36)^. By leveraging students' natural communication preferences, educators can bridge the gap between students' everyday interactions and formal academic requirements^(37)^.

### The role of teachers in anticipating social dynamics in learning interactions

Research findings on Generation Z's language attitudes influence classroom learning dynamics significantly. Sociolinguistic interaction theory asserts that speech forms and strategies in classroom interactions can reflect social relations and familiarity between students and teachers^(38)^. Generation Z's more relaxed and informal language attitudes, such as slang or mixed languages, create an egalitarian interaction atmosphere. However, this interaction can affect the effectiveness of formal learning communication. The success of communication in learning depends on the appropriateness of the language form to the academic context^(39)^. It implies that inappropriate language varieties can obscure the message of learning.

This phenomenon occurs because Generation Z often uses communication patterns influenced by social media, which demand quick, relaxed, humorous, or metaphorical responses. This communication style reflects a shift in interaction values ​​from hierarchical to more egalitarian^(40)^. In the context of learning, this habit makes students prioritize comfort over formality. Although it provides an opportunity to create a more fluid and inclusive learning atmosphere, this pattern also challenges teachers to balance communication flexibility and the required academic formality.

The dynamics of learning influenced by Generation Z's language attitudes require proper management so that relaxed interactions do not compromise the quality of education. Teachers need to be flexible but still maintain formal standards in learning. Thus, students' communication skills reflect their personalities and meet broader academic and social needs. Innovative and contextual teaching strategies can help create dynamic and integrated learning. To maintain the quality of education, teachers must develop adaptive communication strategies. Teachers must educate students about the importance of using appropriate language ​​in context, especially in formal situations such as academic learning. Teachers can conduct this approach through modeling, namely, providing examples of the formal language to students while utilizing student communication media to instill formal values. The learning approach can create adaptive communication patterns without eliminating students' unique characteristics^(41)^.

Teachers must be responsive to students' language attitudes to anticipate the constraints of learning activities. Students' language style that is open and informal responses to teacher questions can be language teaching materials to direct students’ conversations to a more structured academic realm. Language learning materials utilizing students’ speech errors can raise students' awareness of their mistakes and improve their language skills to deliver arguments more systematically and in-depth^(42)^. Therefore, understanding Generation Z's language attitudes in responding to questions increases teachers' insight to create more productive interactions and strengthen learning processes that are more inclusive and relevant to students' learning styles^(43)^.

The social dynamics in the classroom, as seen from the communication patterns of Generation Z, show a change in learning interactions. This finding reinforces the social interaction theory that social and cultural contexts influence learning^(44)^. Generation Z, who speak informal language varieties, reflects the characteristics of a more egalitarian and fluid modern society^(45)^. Although this communication pattern promotes familiarity, the informal language used in the academic context can hinder students' understanding of the formality of communication required in formal learning.

Generation Z spends most of their time communicating online with people they interact with frequently in their daily lives^(46)^. This habit influences their language attitudes in quick responses and is often full of humor or sarcasm. This phenomenon reflects a change in communication culture that is more horizontal and non-hierarchical. In the classroom context, students' speaking styles challenge teachers to balance the need for familiarity with the demands of academic formality. Classroom communication patterns require teachers to adopt a responsive and adaptive approach to students' needs without sacrificing larger learning goals^(47)^. Thus, understanding these social dynamics can help teachers create teaching methods that connect to students' communication styles.

Factors such as egalitarian relationships between teachers and students, the use of local languages, and the influence of social media are determinants of communication patterns in the classroom. Informal language in learning reflects students' efforts to bring their social identities into the learning context^(22)^. While this supports a more inclusive learning atmosphere, teachers need to ensure that these social dynamics are always under control so as not to disrupt the ethics of academic communication. Therefore, a culture-based and technology-based teaching approach directs teachers to combine formality and familiarity in classroom interactions.

In dealing with these social dynamics, teachers must create learning strategies by integrating inclusive and contextual language education approaches. Academic communication training focused on contextual awareness can improve students' ability to adapt to various communication situations^(48)^. This training includes modeling or providing examples of appropriate language use by teachers in learning situations. By understanding these social dynamics, teachers can build dynamic and innovative learning environments to respect the unique characteristics of Generation Z and maintain high academic standards.

## CONCLUSION

This section concludes that in the classroom communication, Generation Z students use informal language forms, slang, local language, and mixed language. They use language strategies including short speech, avoidance, challenges, humor, metaphorical allusions, direct speech, contradictory statements, and sarcasm. The language attitudes are influenced by egalitarian teacher-student relationships, the habit of using regional languages ​​in everyday life, and the influence of interactions on social media. These language attitudes create more egalitarian interactions between students and teachers while also bringing challenges in maintaining academic formality. This phenomenon shows the importance of the teacher's role in balancing familiarity with formal communication education so that students understand the context and language ethics that are appropriate in learning situations. These findings emphasize the need for a teaching approach responding to the communication characteristics of Generation Z. Teachers can utilize their understanding of students' communication patterns to create dynamic and inclusive learning without sacrificing academic standards.
